# Restoring Metabolic Architecture in the South Asian Thin-Outside-Fat-Inside (TOFI) Phenotype: A Mechanistic Rationale for Combining Abscisic Acid and β-Aminoisobutyric Acid

**DOI:** 10.7759/cureus.117275

**Published:** 2026-10-01

**Authors:** Anshu Bhutani, Nitin Patankar, Amitabh Parti, Ketan Pakhale, Anshool Sanghavi, Debasis Basu

**Affiliations:** 1 Endocrinology and Diabetes, Bhutani Hospital and Diabetic Centre, Rohtak, IND; 2 Internal Medicine, Mahatma Gandhi Memorial Medical College, Vashi, IND; 3 Internal Medicine, Fortis Memorial Research Institute, Gurgaon, IND; 4 Internal Medicine, Metabol-Lifestyle Clinic for Metabolic Syndrome, Mumbai, IND; 5 General Medicine, Mahatma Gandhi Memorial Medical College, Vashi, IND; 6 General Medicine, Metta Care Clinic, Kolkata, IND

**Keywords:** abscisic acid, body composition, endogenous glp-1, lancl2, sarcopenic obesity, thin-fat phenotype, uncoupling protein-1, β-aminoisobutyric acid

## Abstract

A large proportion of South Asians exhibit a thin-outside-fat-inside (TOFI) phenotype, also termed metabolically obese normal-weight (MONW); the two terms are used interchangeably in this review. The phenotype combines a normal or near-normal body mass index (BMI) with excess visceral white adipose tissue (WAT), low skeletal muscle mass, low bone mineral density (BMD), and early decline in β-cell function. Glucagon-like peptide-1 receptor agonists (GLP-1 RAs), the dominant pharmacological paradigm for obesity and type 2 diabetes (T2D), may be imperfectly matched to this phenotype. They reduce total body weight effectively, but absolute lean mass declines and BMD may fall during treatment. Their obesity indications are also anchored to BMI thresholds that many TOFI patients do not meet, and their use is constrained in frail older adults and in women planning pregnancy. In this narrative review, we examine the pathophysiology of the TOFI phenotype and develop a mechanistic hypothesis for combining two endogenous human metabolites, abscisic acid (ABA) and β-aminoisobutyric acid (BAIBA). The aim of this combination would be to shift the therapeutic target from weight loss toward restoration of body composition and metabolic function. In predominantly preclinical studies, BAIBA, an exercise-induced myokine, promotes uncoupling protein-1 (UCP-1)-dependent browning of WAT through peroxisome proliferator-activated receptor alpha (PPARα). It also supports skeletal muscle through adenosine monophosphate-activated protein kinase (AMPK)-PPARδ signalling and protects osteocytes through Mas-related G protein-coupled receptor member D (MRGPRD). In preclinical and small early human studies, ABA, acting through lanthionine synthetase C-like protein 2 (LANCL2), has been shown to stimulate GLP-1 secretion and to promote insulin-independent, glucose transporter type 4 (GLUT4)-mediated glucose uptake in skeletal muscle. The two molecules appear mechanistically complementary: BAIBA targets fat quality and musculoskeletal preservation, whereas ABA targets glucose disposal independent of β-cell secretory capacity. We propose a conceptual three-pillar model and summarise the evidence for each component according to evidence level. We also review preliminary data from a 12-week, open-label, single-arm, investigator-led cohort of 30 adults with obesity, in which mean body weight fell by approximately 8%, with no serious adverse events, hypoglycaemic episodes or discontinuations reported. No randomised controlled trial of the combination has yet been published. The concept is hypothesis-generating and requires confirmation in randomised controlled trials in South Asian populations, with body-composition, glycaemic and β-cell function endpoints.

## Introduction and background

The clinical management of obesity and type 2 diabetes (T2D) has historically equated therapeutic success with a falling number on the scale. This weight-centric model assumes that fat loss, and the lean-mass loss that usually accompanies it, is an acceptable trade-off. For a meaningful proportion of patients, and for South Asians in particular, that assumption may be unsafe, because many begin treatment with limited muscle reserve; sarcopenia is already common among older Indian adults [1]. We therefore suggest that the relevant clinical endpoint is not weight alone but body composition and metabolic function: selective reduction of dysfunctional white adipose tissue (WAT), preservation of skeletal muscle and bone, and improved glycaemic control. Skeletal muscle is the largest site of insulin-stimulated glucose disposal and a principal determinant of basal metabolic rate. Low muscle mass is associated with weight regain and with frailty, a state of reduced physiological reserve and increased vulnerability to falls, disability and death. When low muscle mass coexists with excess adiposity, the resulting sarcopenic obesity carries a higher risk of adverse outcomes than either condition alone [1,2].

South Asians display a distinctive body composition, described as the "Asian-Indian" or "thin-fat" phenotype and also termed thin-outside-fat-inside (TOFI) or metabolically obese normal-weight (MONW). In this review, TOFI and MONW are used interchangeably to describe individuals with a normal or near-normal body mass index (BMI) who nonetheless carry excess visceral adiposity, low skeletal-muscle mass and an elevated cardiometabolic risk profile. At any given BMI, South Asians carry more visceral fat, less skeletal muscle and a greater burden of insulin resistance than European-ancestry populations. They also develop dysglycaemia at lower anthropometric thresholds and show poorer β-cell compensation [3-5]. In India, the ICMR-INDIAB national survey reported a prevalence of 11.4% for diabetes and 15.3% for prediabetes, corresponding to an estimated 101 million and 136 million adults, respectively. The same survey found generalised obesity in 28.6% and abdominal obesity in 39.5% of adults [6]. These figures sit against a backdrop of widespread physical inactivity, with more than half of Indian adults classified as inactive [7]. A large fraction of metabolically unhealthy Indians are not captured by Western BMI-based obesity definitions. This has direct consequences for any therapy whose regulatory label is anchored to a high BMI.

Against this background, the incretin-based glucagon-like peptide-1 receptor agonists (GLP-1 RAs) and the dual glucose-dependent insulinotropic polypeptide (GIP)/GLP-1 RAs have transformed pharmacological weight management [8,9]. Their efficacy for total weight reduction is not in dispute. The pivotal programmes also included dual-energy X-ray absorptiometry (DXA) body-composition substudies. These showed that most of the weight lost was fat mass and that lean mass as a proportion of body weight was relatively preserved [8,9]. The questions this review raises are therefore narrower and population-specific, and they concern how the accompanying absolute loss of lean and bone mass applies to patients who start with limited reserve. The TOFI phenotype is defined by pre-existing low lean mass, low bone density, and an early decline in β-cell function. In such patients, is the right physiological match a therapy that further reduces lean and bone mass, acts through an insulin-dependent axis with a finite ceiling, and is licensed for a BMI range that most of them never reach? We suggest that it often may not be. On that basis, we develop a mechanistic hypothesis for an alternative, body-composition-preserving approach that combines two endogenous human metabolites, abscisic acid (ABA) and β-aminoisobutyric acid (BAIBA). Because clinical evidence for this combination is currently limited to preliminary open-label data, the concept is presented as real-world evidence-generating and requires confirmation in randomised controlled trials.

This article is a narrative, mechanism-focused review rather than a systematic review, and the Preferred Reporting Items for Systematic Reviews and Meta-Analyses (PRISMA) methodology was therefore not applied. PubMed/MEDLINE and Google Scholar were searched for English-language articles using combinations of the terms thin-fat, thin-outside-fat-inside, metabolically obese normal weight, South Asian, body composition, sarcopenia, bone mineral density (BMD), β-cell function, GLP-1 RA, semaglutide, tirzepatide, lean mass, UCP1, BAIBA, and ABA. The reference lists of key articles were also hand-searched. Studies were selected by author judgement based on the mechanistic relevance and applicability to South Asian populations. Randomised trials, meta-analyses, and large cohort studies were prioritised for clinical statements, and primary mechanistic studies for pathway statements. Findings were grouped thematically (TOFI phenotype, incretin therapy outcomes, BAIBA biology, ABA biology, and combination rationale) and synthesised narratively. No formal risk-of-bias assessment or quantitative pooling was performed.

## Review

Metabolic architecture of the TOFI phenotype

Dysfunctional WAT and Impaired Thermogenesis

Visceral WAT is the proximate driver of the phenotype. Expanded, dysfunctional visceral adipose tissue exports free fatty acids and pro-inflammatory adipokines, promotes ectopic lipid deposition in liver and muscle, and sustains systemic insulin resistance [3]. Uncoupling protein-1 (UCP-1), the inner-mitochondrial-membrane protein responsible for non-shivering thermogenesis, dissipates the proton gradient as heat rather than adenosine triphosphate. It is essential for adaptive thermogenesis and for the substrate-oxidising capacity of brown and "beige" adipocytes [10]. Common UCP1 promoter variants, most notably the -3826A/G polymorphism, have been associated with reduced UCP1 messenger RNA expression, lower resting energy expenditure, higher adiposity, increased T2D risk and a blunted response to weight-loss interventions [11-14]. A meta-analysis supports an association between UCP1 variants and obesity that is most evident in Asian populations [15]. These data derive from broader Asian cohorts with obesity or T2D rather than from individuals specifically characterised as TOFI or MONW, and genotype-specific evidence in the TOFI phenotype is currently lacking. A reduced thermogenic reserve could nonetheless favour energy storage over dissipation in carriers of these variants, representing a potential genetic headwind against purely energy-restriction approaches.

Low Skeletal-Muscle Reserve and Accelerated Sarcopenia

DXA-based comparisons have shown that South Asians have lower lean mass than European-ancestry adults of similar BMI [16]. In a large United Kingdom cohort, South Asians also had lower handgrip strength, a functional measure that was associated with a higher prevalence of diabetes [17]. The deficit therefore extends to muscle strength and quality as well as mass, which is relevant because current sarcopenia definitions prioritise muscle strength and physical performance alongside muscle mass. Lean mass is further lost with age, physical inactivity, relatively low androgenicity and dysglycaemia [3,7,18]. Anabolic signalling through the phosphoinositide 3-kinase (PI3K)/protein kinase B (AKT) axis is a principal determinant of muscle hypertrophy, and oxidative capacity is supported by peroxisome proliferator-activated receptor delta (PPARδ) signalling [19]. Because skeletal muscle is also the largest site of insulin-stimulated glucose disposal, a low muscle reserve compounds both hyperglycaemia and weight-loss resistance. In this setting, any intervention that subtracts further from muscle mass risks converting a thin-fat phenotype into overt sarcopenic obesity, a state of higher morbidity, frailty and mortality whose prevalence is already high in older Indians [1,2].

Low Bone Mineral Density (BMD)

Osteoporosis and osteopenia are highly prevalent in India and present at younger ages than in Western cohorts, with a substantial burden among peri- and post-menopausal women [20,21]. Population-based studies in Indian adults have reported a high prevalence of low BMD from the fifth decade onward [22]. Widespread vitamin D deficiency and low dietary calcium intake contribute materially to this deficit [23]. Estrogen withdrawal accelerates osteocyte apoptosis and bone resorption and frequently coexists with sarcopenia, while physical inactivity removes the mechanical-loading signal that maintains bone [2,24]. With ageing, osteocytes also show reduced expression of Mas-related G protein-coupled receptor member D (MRGPRD), a receptor that mediates protective signalling from muscle to bone [25]. A phenotype already biased toward low BMD is poorly tolerant of any therapy that further accelerates bone loss during rapid weight reduction.

Early Decline in β-Cell Function

South Asians and East Asians characteristically exhibit early and pronounced loss of β-cell function relative to their degree of insulin resistance, with a large fraction of secretory capacity already compromised by the time T2D is diagnosed [4,5,26]. Therapies that achieve glycaemic control predominantly by amplifying an insulin-dependent secretory axis therefore confront a structural ceiling in this population, because the cell being asked to work harder is already failing. This limitation bears directly on incretin therapy, whose glucose-lowering action depends on residual β-cell function. In T2D, markers of β-cell failure such as low fasting C-peptide predict a poorer glycaemic response to GLP-1 RAs [27]. A glucose-disposal route that does not depend on β-cell output is therefore mechanistically attractive.

Table 1 summarises these four structural deficits, the mechanistic basis of each and the therapeutic implication that follows from it. Figure 1 illustrates how the same four deficits are distributed across adipose tissue, skeletal muscle, the bone-muscle axis and the pancreatic β-cell, and how physical inactivity amplifies each of them so that all four converge on frailty.

**Table 1:** Structural deficits of the TOFI phenotype and their therapeutic implications TOFI: thin-outside-fat-inside; WAT: white adipose tissue; UCP-1: uncoupling protein-1; UCP1: uncoupling protein-1 gene; T2D: type 2 diabetes Source: dysfunctional visceral WAT and low thermogenic reserve [3,10-15]; low skeletal muscle mass and strength [3,7,16-19]; low bone mineral density [2,20-25]; early decline in β-cell function [4,5,26,27]

Structural deficit	Mechanistic basis	Therapeutic implication
Dysfunctional visceral WAT; low thermogenic reserve	UCP1 promoter variants (e.g. -3826A/G; data from broader Asian cohorts) → lower UCP1 expression	Favour WAT-selective loss via UCP-1 activation/browning, not generalised weight loss
Low skeletal-muscle mass	Lower baseline lean mass; inactivity, low androgenicity and dysglycaemia accelerate loss	Preserve/anabolise muscle; avoid catabolic therapies
Low bone mineral density	High osteopenia/osteoporosis prevalence; estrogen loss; low mechanical loading	Protect osteocytes; avoid accelerating bone loss
Early decline in β-cell function	Marked loss of secretory capacity relative to insulin resistance before T2D diagnosis	Use insulin-independent glucose disposal; relieve β-cell load

**Figure 1:**
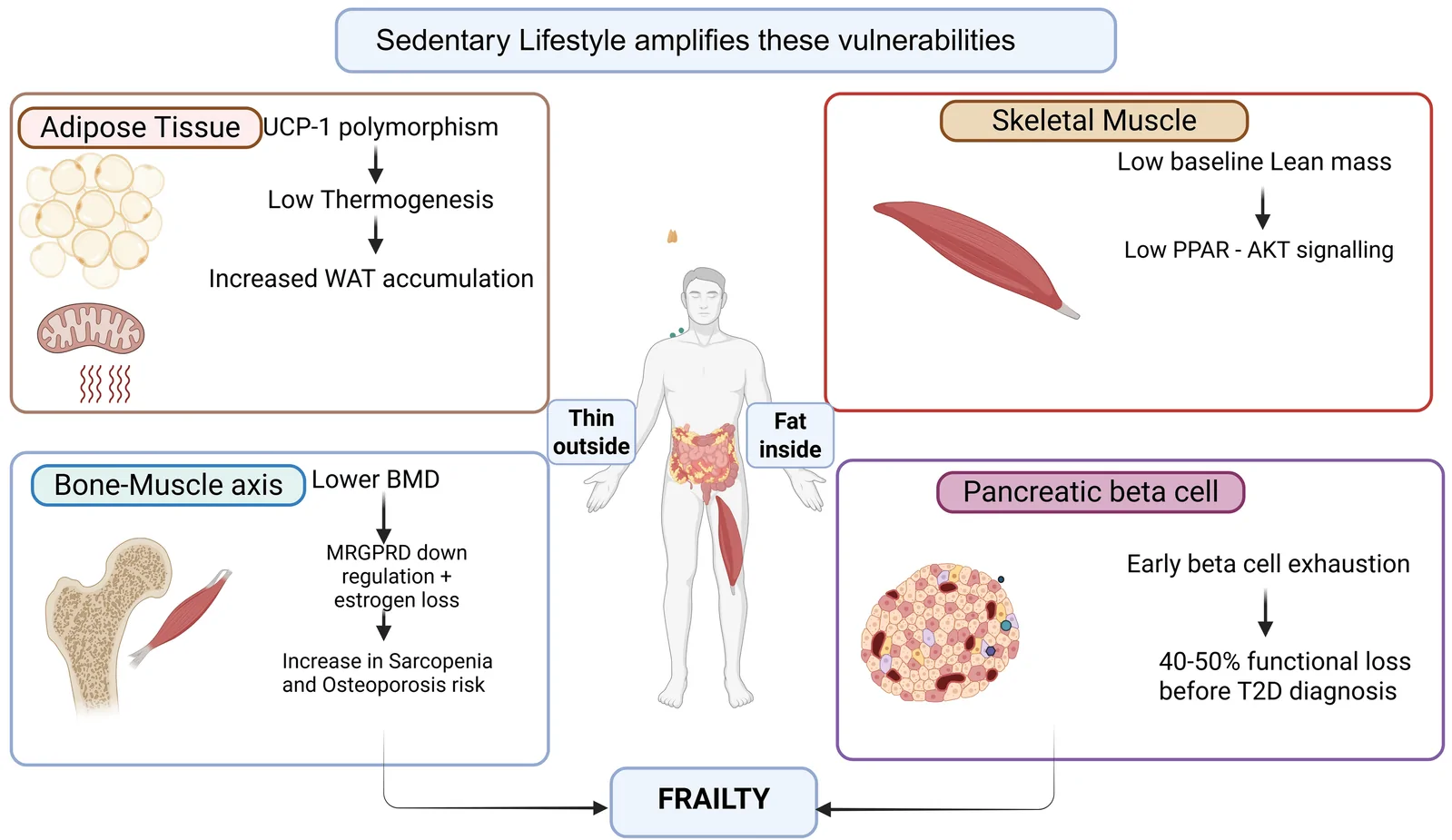
Structural deficits of the thin-outside-fat-inside phenotype in South Asian Indians TOFI: thin-outside-fat-inside; UCP-1: uncoupling protein-1; WAT: white adipose tissue; MRGPRD: Mas-related G protein-coupled receptor member D; PPARδ: peroxisome proliferator-activated receptor delta; T2D: type 2 diabetes; DXA: dual-energy X-ray absorptiometry; HOMA-B: homeostasis model assessment of β-cell Four structural deficits characterise the TOFI phenotype at normal or low body mass index. In adipose tissue, low-expression UCP1 promoter variants, identified in broader Asian cohorts with obesity or T2D rather than in TOFI individuals specifically, are associated with blunted thermogenesis and WAT accumulation [11-15]. In skeletal muscle, baseline lean mass and strength are low [3,7,16-18], with potential attenuation of PPARδ and protein kinase B (AKT) signalling, which support oxidative capacity and muscle growth [19]. Along the bone-muscle axis, age-related downregulation of MRGPRD, estrogen withdrawal, and vitamin D and calcium insufficiency reduce osteocyte survival and bone mineral density [20-25]. In pancreatic β-cells, secretory capacity is substantially lost before T2D is diagnosed [4,5,26]. Physical inactivity amplifies each deficit, and all four converge on frailty. Each deficit can be assessed clinically: visceral adiposity by waist circumference, DXA or magnetic resonance imaging; lean mass by DXA or bioelectrical impedance, with muscle strength by handgrip dynamometry; bone by DXA-derived bone mineral density; and β-cell function by fasting C-peptide or the HOMA-B function. Solid arrows indicate the direction of the causal sequence described within each panel, and the arrows converging on the lower centre of the figure indicate that all four deficits contribute to frailty. Image credit: created by the authors using BioRender (2026); https://BioRender.com/goph3ew

Why the incretin paradigm is an imperfect match for the TOFI phenotype

​​​​​​The four structural deficits described above determine how any pharmacotherapy should be judged in this population. GLP-1 RAs and dual agonists act principally by enhancing glucose-dependent insulin secretion, suppressing glucagon, slowing gastric emptying and reducing appetite, and they are highly effective at reducing total body weight [28]. In the pivotal semaglutide treatment effect in people with obesity (STEP) and SURMOUNT programmes, mean reductions ranged from roughly 10% to over 20% of baseline weight [8,9]. The concern for the TOFI phenotype is therefore not whether weight falls, but what tissue is lost, in whom, and what happens afterwards.

Composition of the Weight Lost and the Response Ceiling

Body-composition substudies of the pivotal trials show that absolute lean (fat-free) mass declines during GLP-1 RA and dual-agonist therapy. Lean-mass loss is commonly reported as approximately 25% to 40% of total weight lost across drugs, doses, and durations [8,9]. Because fat mass falls faster, lean mass expressed as a proportion of body weight is often preserved or even increased, and physical performance is frequently maintained [9]. We therefore adopt the balanced reading that absolute lean mass declines on therapy and that whether this is clinically benign depends largely on the patient's baseline reserve. In a European-ancestry adult with abundant muscle, a modest absolute loss may be inconsequential. In a South Asian patient who is already near a sarcopenic threshold, the same absolute loss may not be. Response is also not uniform. A meta-analysis of 21,023 patients across 33 randomised trials found that a substantial fraction did not achieve 5% to 10% weight loss, with markedly higher non-response among patients with established T2D [29]. This pattern may partly reflect the limits of an insulin-dependent axis in patients with reduced β-cell reserve.

Bone

Weight loss during incretin therapy has been accompanied by reductions in BMD and, in some analyses, by increased bone-resorption markers. In a randomised, placebo-controlled trial in 64 adults at increased fracture risk, 52 weeks of semaglutide 1.0 mg lowered areal BMD, measured by DXA, relative to placebo at the lumbar spine (estimated treatment difference -0.018 g/cm²) and total hip (-0.020 g/cm²), and increased the bone-resorption marker C-terminal telopeptide of type I collagen [30]. A retrospective cohort, reported as a conference abstract, associated greater weight loss on semaglutide or tirzepatide with greater decline in hip BMD [31]. Volumetric BMD and bone-strength data are largely unavailable. Whether fracture risk is increased remains uncertain: meta-analyses of randomised trials have not shown a consistent increase in fractures with GLP-1 RAs, although these trials were neither designed nor powered for fracture outcomes [32]. These findings nonetheless apply to a population with a high baseline prevalence of osteopenia and osteoporosis and accelerated peri-menopausal BMD loss [20,21]. Real-world incretin use also increasingly extends to two years or more, so the potential cumulative skeletal cost warrants specific study in South Asians.

The Lean-BMI, Metabolically Obese Segment

A large fraction of metabolically unhealthy Indians have a normal or near-normal BMI and therefore fall outside the BMI range for which anti-obesity pharmacotherapy is licensed [3,6]. For these patients, a weight-loss-centric therapy is both off-label and conceptually mismatched. Further lowering an already modest body weight could accelerate muscle loss and worsen the sarcopenic-obese trajectory the clinician is trying to prevent. The therapeutic need in this group is qualitative, relating to fat quality, glucose disposal and musculoskeletal integrity, rather than quantitative in kilograms.

Reproductive-Age Women, Tolerability, Neuromuscular Integrity, and Frailty

GLP-1 RAs are not recommended during pregnancy, and product labelling advises discontinuation before a planned conception, at least two months beforehand in the case of semaglutide. This constrains their use in the large population of women with polycystic ovary syndrome (PCOS)-related subfertility [33-35]. Gastrointestinal intolerance is a principal driver of discontinuation, and real-world persistence is limited. Most of the weight lost is regained after treatment stops [36,37]. If regained weight is disproportionately fat, as has been reported after diet-induced weight loss [38], discontinuation could leave body composition less favourable than at baseline. Frailty was neither an entry criterion nor a measured outcome in the pivotal obesity trials, whose mean participant age was in the mid-forties [8]. In an observational study, older adults with T2D treated with semaglutide showed declines in grip strength, gait speed and physical-performance scores, alongside biomarker evidence of neuromuscular-junction degradation [39]. This is a particular concern where muscle reserve is already low [1]. Recognition of this issue is growing. In a randomised phase 2 trial, adding the activin type II receptor antibody bimagrumab to semaglutide largely preserved lean mass while augmenting fat loss [40], and the selective androgen receptor modulator enobosarm has been evaluated alongside semaglutide in a registered phase 2 trial [41]. Table 2 sets out representative weight outcomes reported for these agents, together with the body-composition consideration attached to each.

**Table 2:** Representative weight outcomes and body-composition considerations for incretin therapies Δ: change from baseline; BMD: bone mineral density; DXA: dual-energy X-ray absorptiometry; STEP: semaglutide treatment effect in people with obesity Values are approximate and drawn from the cited source material [8,9,30,31,37]. They are intended for orientation only and are not head-to-head comparisons; populations, treatment durations, and body-composition methods differ across studies

Trial/source	Mean weight Δ	Agent	Body-composition consideration
STEP 1 [8]	≈ -15%	Semaglutide 2.4 mg	Absolute lean-mass loss ≈25-40% of total weight lost; lean mass as a proportion of body weight relatively preserved
SURMOUNT-1 (body-composition substudy) [9]	≈ -21%	Tirzepatide 15 mg	Greatest total loss; absolute lean-mass reduction with relative proportional preservation
STEP 1 extension [37]	Regain after stop	Semaglutide 2.4 mg	Substantial regain after discontinuation
Fracture-risk randomised controlled trial and retrospective BMD cohort [30,31]	-	Semaglutide; tirzepatide	Lower areal BMD (DXA) at spine and hip; greater weight loss associated with greater hip-BMD decline; fracture effect uncertain

Reframing the target: restoring metabolic architecture

The limitations described above suggest that, in the TOFI phenotype, the therapeutic target should shift from weight loss to restoration of metabolic architecture. We propose a conceptual framework of three actions pursued together rather than sequentially. The first pillar is WAT-selective loss, in which thermogenic, fat-dominant loss is promoted by activating UCP-1-dependent browning. This could potentially offset the reduced thermogenic capacity associated with low-expression UCP1 polymorphisms. The second pillar is musculoskeletal preservation. Here, skeletal muscle is supported through adenosine monophosphate-activated protein kinase (AMPK)-PPARδ signalling and bone through osteocyte survival, so that both tissues are maintained while fat mass is lost. The third pillar is β-cell-sparing glucose control. Glucose disposal is improved through an insulin-independent route involving AMPK and glucose transporter type 4 (GLUT4), and endogenous incretin tone is supported, relieving rather than overdriving the β-cell. Within the combination proposed below, BAIBA is expected to act primarily on the first and second pillars and ABA primarily on the third. Figure 2 depicts these three pillars and shows how, engaged together, they may converge on an integrated metabolic benefit.

**Figure 2:**
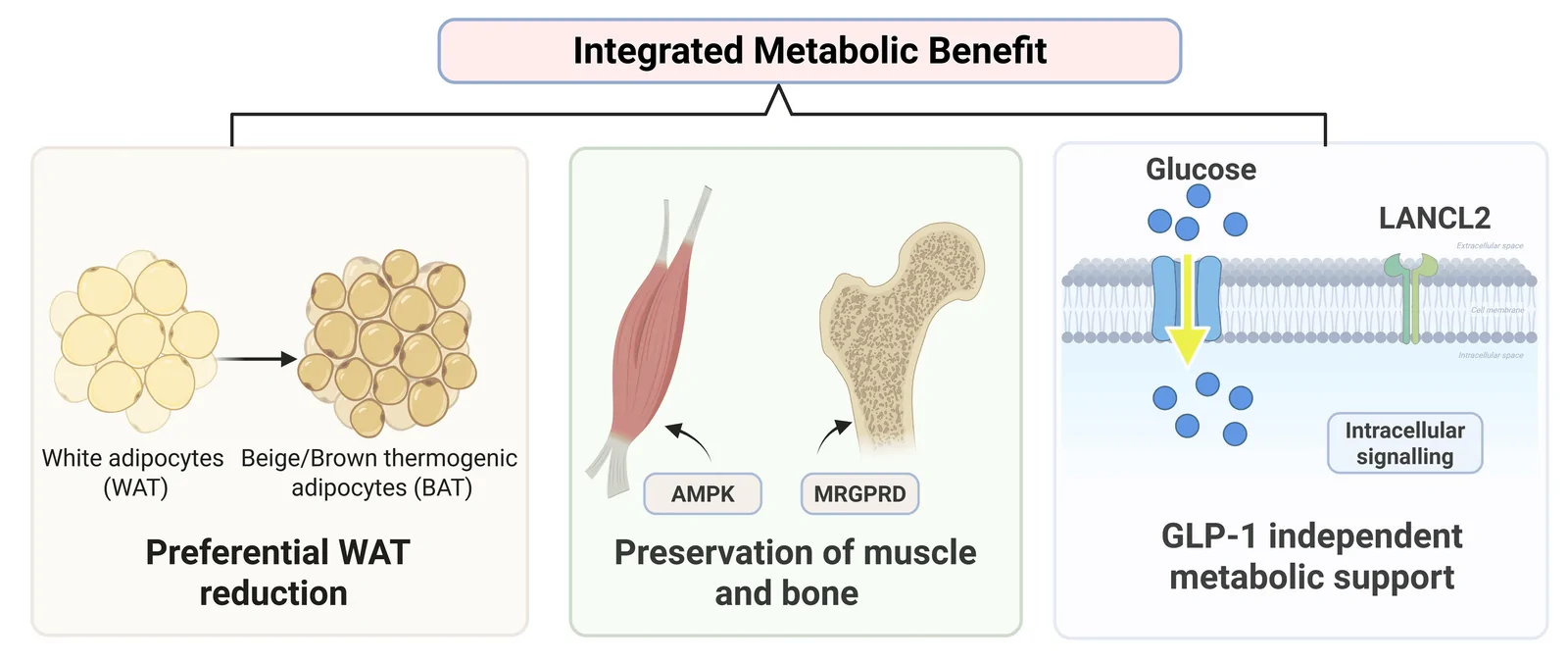
The three-pillar model for restoring metabolic architecture TOFI: thin-outside-fat-inside; UCP-1: uncoupling protein-1; WAT: white adipose tissue; AMPK: adenosine monophosphate-activated protein kinase; MRGPRD: Mas-related G protein-coupled receptor member D; LANCL2: lanthionine synthetase C-like protein 2x; BAIBA: β-aminoisobutyric acid; ABA: abscisic acid; BAT: brown adipose tissue; GLP-1: glucagon-like peptide-1 Three therapeutic pillars are proposed for the TOFI phenotype. The first pillar is preferential WAT reduction via UCP-1-mediated browning. The second pillar is preservation of muscle through AMPK signalling and of bone through MRGPRD signalling. The third pillar is LANCL2-mediated, insulin-independent glucose disposal that bypasses the pancreatic β-cell. Engaged together, the three pillars may deliver an integrated metabolic benefit [25,42-45]. The model is conceptual; BAIBA is proposed to act primarily on the first and second pillars and ABA on the third. Solid arrows indicate the direction of the conversion or signalling step described. Image credit: created by the authors using BioRender (2026); https://BioRender.com/adbhszg

Because the target is metabolic architecture rather than body weight, the outcomes by which it should be judged differ from those of conventional weight-management trials. Glycaemic control would be assessed using glycated haemoglobin (HbA1c), fasting plasma glucose and fasting insulin. Insulin resistance and β-cell function would be assessed using the homeostasis model assessment indices (HOMA-IR and HOMA-B), fasting C-peptide, or an oral glucose tolerance test-derived disposition index. Visceral adiposity and lean mass would be measured by DXA or magnetic resonance imaging, for example, visceral adipose tissue area and appendicular lean mass index. Bone would be assessed by DXA-derived BMD and bone turnover markers, and muscle function by handgrip strength and physical-performance tests. Body weight remains a useful secondary measure. Within this framework, however, a modest weight change accompanied by reduced visceral fat, preserved lean mass and improved glycaemia would represent a favourable outcome. This three-pillar model and its associated measures are proposed as a conceptual framework to guide trial design; they have not yet been validated as a composite clinical endpoint, and such validation will require prospective controlled studies. To our knowledge, no approved agent has been shown to address all three pillars concurrently in this population. The central hypothesis of this review is that two endogenous human metabolites may do so in combination.

β-Aminoisobutyric acid (BAIBA): a myokine linking fat, muscle, and bone

BAIBA is a small-molecule myokine generated endogenously as a catabolite of thymine (the R-enantiomer) and valine (the L-enantiomer). It is secreted by skeletal muscle in response to exercise and peroxisome proliferator-activated receptor gamma coactivator 1-alpha (PGC-1α) signalling and can be regarded as an endogenous signal that couples muscle activity to systemic substrate handling [42,46]. The evidence summarised below is predominantly preclinical. Human data are limited to observational associations between circulating BAIBA and cardiometabolic or skeletal parameters, and no interventional human studies of BAIBA supplementation on these outcomes have been published.

BAIBA and White-Fat Browning

In the defining study, Roberts et al. identified BAIBA as a PGC-1α-regulated myokine that increases expression of brown-adipocyte-specific genes, including UCP1 and cell death-inducing DNA fragmentation factor-like effector A (CIDEA), in white adipocytes. It also induced hepatic β-oxidation and conferred a brown-adipose-like phenotype on human pluripotent stem cell-derived adipocytes in vitro. In mice, BAIBA improved glucose homeostasis, with dose-dependent increases in UCP-1 expression and a reduction in white-fat content, through a mechanism dependent on peroxisome proliferator-activated receptor alpha (PPARα) [42]. In the same report, plasma BAIBA concentrations in human cohorts were inversely associated with cardiometabolic risk factors, an observational association [42]. For the TOFI phenotype, the relevance of these findings is that BAIBA acts as a transcriptional inducer of the thermogenic programme. It may therefore augment UCP1 expression even in carriers of low-expression UCP1 promoter variants, although this has not been tested in genotyped individuals. This rationale is consistent with the broader interest in UCP-1 activation and adipose browning as anti-obesity targets [47,48].

BAIBA and Skeletal Muscle

Beyond its endocrine action on fat, BAIBA has autocrine effects on muscle. In cultured myocytes and in mice fed a high-fat diet, BAIBA attenuated palmitate- and diet-induced insulin resistance and inflammation through an AMPK-PPARδ-dependent pathway, supporting fatty-acid oxidation [43]. This is the same axis engaged by pharmacological "exercise mimetics" in mice [49]. In middle-aged mice, L-BAIBA combined with voluntary wheel running enhanced musculoskeletal properties, consistent with an action that supports muscle mass and function [50]. For a phenotype defined by low muscle reserve, a molecule that may both promote browning of fat and support muscle is mechanistically aligned with the therapeutic goal, although these effects remain to be demonstrated in humans.

BAIBA and Bone

Kitase et al. identified L-BAIBA as a muscle-derived osteocyte-survival factor [25]. In osteocyte-like cells in vitro, L-BAIBA acting through MRGPRD protected against reactive-oxygen-species-induced apoptosis by preserving mitochondrial integrity. Under the conditions tested, its protective effect was reported to be comparable to or greater than that of estrogen or N-acetylcysteine. In a murine hindlimb-unloading model, L-BAIBA prevented loss of bone and muscle function [25,51]. This estrogen-independent route to osteocyte protection is of potential relevance to peri- and post-menopausal women. In ovariectomised mice, exercise-induced L-BAIBA suppressed osteoclastogenesis, and in women with postmenopausal osteoporosis, circulating L-BAIBA levels correlated positively with BMD, an observational association [52]. In osteoblastic cells in vitro, BAIBA also promoted proliferation and differentiation [53]. The protective effect declines with age, which has been attributed to reduced MRGPRD expression rather than reduced BAIBA production [25]. This could support supplementation to sustain signalling as endogenous receptor tone falls. It also implies, however, that target engagement may be limited when receptor density is low. Whether higher circulating BAIBA restores signalling in older adults has not been tested and would need to be addressed in dose-finding studies. Figure 3 summarises these three tissue-level axes of BAIBA action across adipose tissue, skeletal muscle and bone.

**Figure 3:**
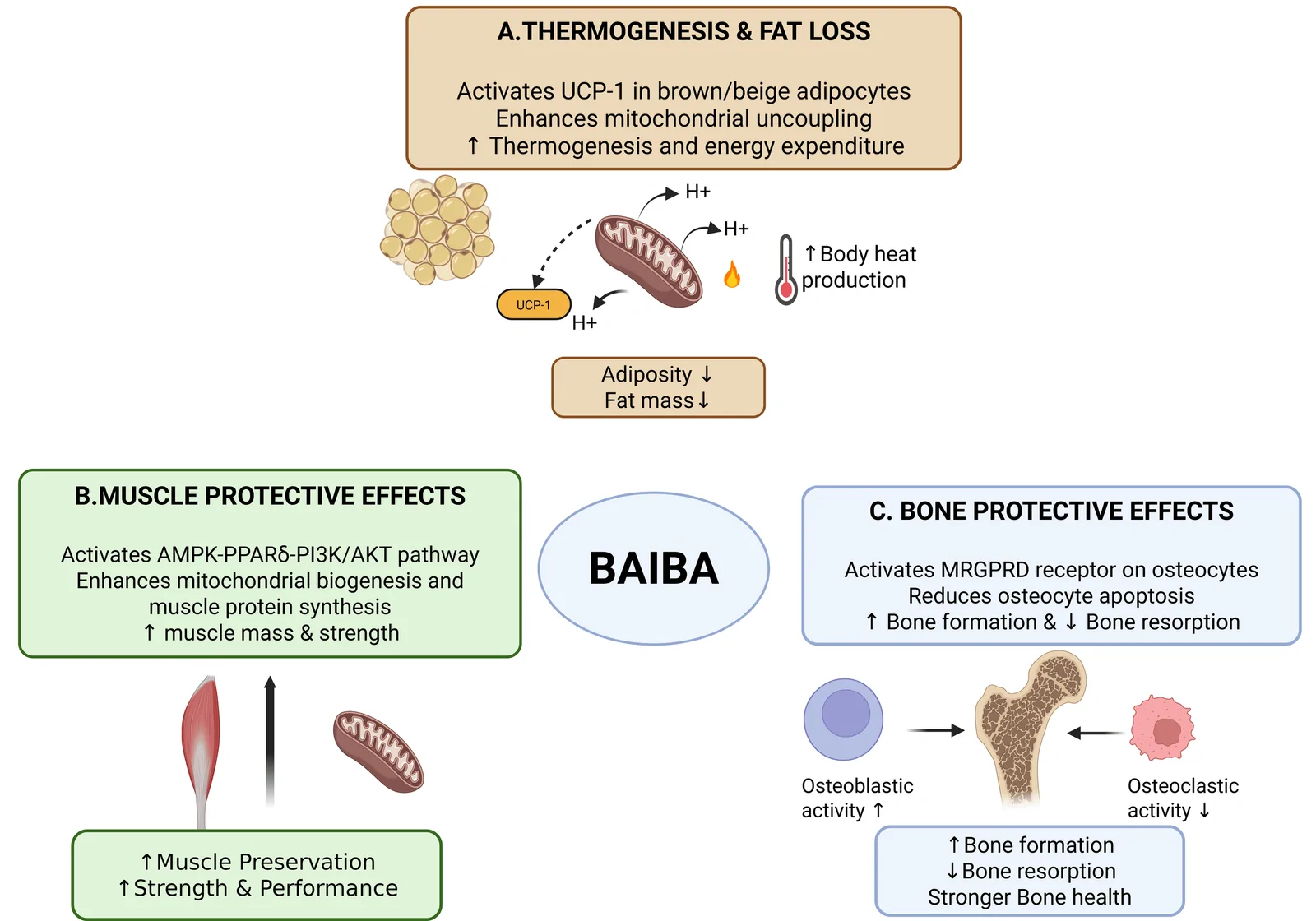
Pleiotropic tissue-level actions of BAIBA on the adipose tissue, skeletal muscle, and bone UCP1: uncoupling protein-1; PPARδ: peroxisome proliferator-activated receptor delta; PI3K: phosphoinositide 3-kinase; AMPK: adenosine monophosphate-activated protein kinase; MRGPRD: Mas-related G protein-coupled receptor member D; BAIBA: β-aminoisobutyric acid Schematic of the three mechanistic axes of BAIBA, an exercise-induced myokine and valine/thymine catabolite. (A) BAIBA induces UCP-1 in brown/beige adipocytes, uncoupling mitochondrial oxidative phosphorylation to raise heat production and lower adiposity [42]. (B) It activates AMPK-PPARδ and PI3K/protein kinase B (AKT) signalling, enhancing mitochondrial biogenesis and muscle protein synthesis to preserve muscle mass and strength [19,43,49,50]. (C) It binds osteocyte MRGPRD and reduces osteocyte apoptosis, increasing osteoblastic and decreasing osteoclastic activity in favour of bone formation [25,51-53]. Panels A-C are based predominantly on in vitro and murine data; human evidence is limited to observational associations between circulating BAIBA and cardiometabolic risk or bone mineral density. Solid arrows indicate direct effects, that is, a step that follows directly from the preceding one within the same pathway, and also indicate the direction of proton movement in panel A and of cellular contribution to bone remodelling in panel C; dashed arrows indicate indirect effects. ↑ increase, ↓ decrease Image credit: created by the authors using BioRender (2026); https://BioRender.com/adbhszghttps://BioRender.com/qgmccas

Abscisic acid (ABA): an endogenous insulin-independent glucose regulator

ABA is an isoprenoid signalling molecule conserved from plants to mammals. In humans, it is produced endogenously by cells including granulocytes and pancreatic β-cells [54], and it acts through the lanthionine synthetase C-like receptor family, principally lanthionine synthetase C-like protein 2 (LANCL2). At nanomolar-to-micromolar concentrations, ABA regulates glycaemia in experimental models through mechanisms that are largely insulin-independent [44]. The human evidence, summarised below, is limited to small, short-term studies.

ABA and GLP-1 Secretion

In enteroendocrine L-cells in vitro, ABA stimulated glucagon-like peptide-1 (GLP-1) secretion through a cyclic adenosine monophosphate (cAMP)/protein kinase A (PKA)-dependent mechanism, and oral ABA raised plasma GLP-1 and insulin in rats [55]. Conversely, GLP-1 enhanced ABA release from β-cells, suggesting a bidirectional ABA-incretin loop [55]. These findings suggest that ABA may act as an endogenous, orally active modulator of the incretin axis, supporting GLP-1 tone physiologically rather than supplying a supraphysiological receptor agonist. Whether this effect is clinically meaningful in humans has not been established.

ABA and Insulin-Independent Muscle Glucose Uptake

The property of ABA most relevant to the TOFI phenotype is its capacity to stimulate skeletal-muscle glucose uptake without insulin. In L6 myoblasts and in mouse muscle imaged by micro-positron emission tomography, ABA increased uptake of glucose analogues in an AMPK-dependent and LANCL2-dependent manner. Pharmacological AMPK inhibition or LANCL2 silencing abolished the effect, and ABA increased phosphorylated AMPK, GLUT4, and PGC-1α expression [44]. LANCL1 was subsequently identified in muscle cells as a second ABA receptor that activates the AMPK/PGC-1α/sirtuin 1 (Sirt1) axis to stimulate glucose transport and mitochondrial respiration [45]. Because this route does not depend on insulin secretion, it could complement insulin-dependent strategies in patients with early β-cell decline. It also engages the AMPK node implicated in muscle and bone integrity, although this link between glycaemic and musculoskeletal effects remains inferential.

Human Evidence and Anti-inflammatory Action

Human studies of ABA have used different preparations, namely, synthetic ABA, ABA-rich fruit extracts and dietary ABA, whose dose and bioavailability may differ substantially, so their results are not directly interchangeable. In the first human study, volunteers who took an ABA-rich fruit extract providing approximately 0.5-0.85 µg ABA/kg body weight with an oral glucose load or with standard meals showed significantly lower glycaemic and insulinaemic responses than without the extract, indicating oral bioavailability and a glucose-lowering effect not dependent on increased insulin secretion; parallel findings were obtained with synthetic ABA and fruit extract in rats [56]. In an uncontrolled before-and-after study, 10 healthy subjects who took an ABA-rich food supplement providing 1 µg ABA/kg body weight daily for 75 days showed significant reductions in fasting blood glucose, HbA1c, total cholesterol, and BMI, with supportive findings in high-glucose-fed mice [57]. In mouse models of diet-induced obesity and in db/db mice, an ABA-enriched fig extract at 0.125 µg ABA/kg improved glucose tolerance and insulin sensitivity, an effect lost when LANCL2 was deleted from skeletal muscle [58]. In murine models, ABA also exerts anti-inflammatory effects through ligand-binding-domain-independent activation of peroxisome proliferator-activated receptor gamma (PPARγ). This may contribute to improved insulin sensitivity in the inflamed adipose milieu of the TOFI phenotype [59]. Figure 4 depicts the LANCL2-mediated signalling that underlies these peripheral and enteroendocrine actions of ABA.

**Figure 4:**
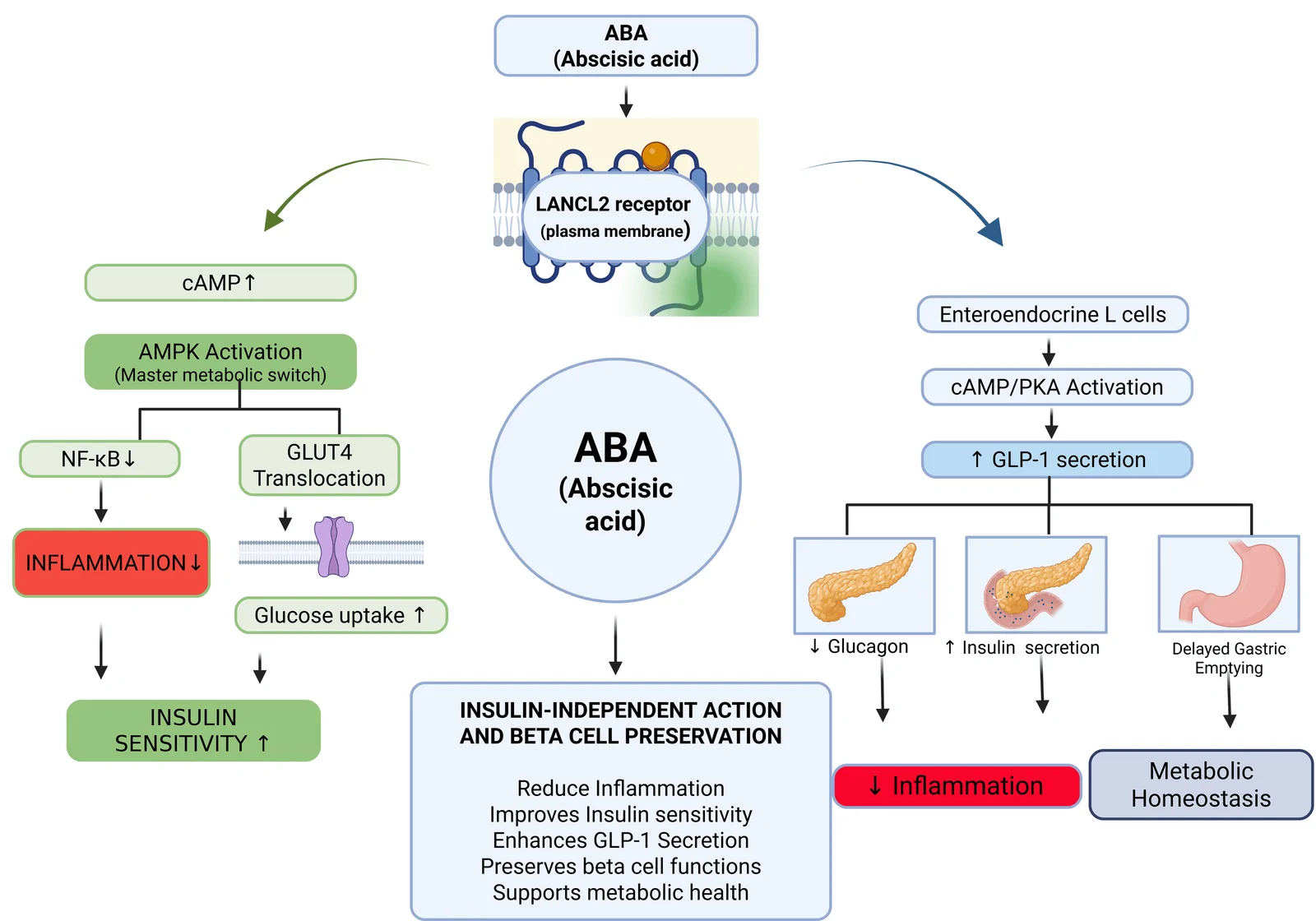
LANCL2-mediated mechanistic actions of ABA on glucose homeostasis, inflammation, and incretin signalling LANCL2: lanthionine synthetase C-like protein 2; AMPK: adenosine monophosphate-activated protein kinase; NF-κB: nuclear factor kappa B; GLUT4: insulin-independent glucose transporter type 4; PKA: protein kinase A; cAMP: cyclic adenosine monophosphate; GLP-1: glucagon-like peptide-1; ABA: abscisic acid Schematic of ABA signalling via the plasma-membrane receptor LANCL2, which raises intracellular cyclic adenosine monophosphate (cAMP) along two convergent arms [44,45]. Peripherally (left), AMPK activation suppresses NF-κB-driven inflammation and drives GLUT4 translocation, improving glucose uptake and insulin sensitivity [44,45,59]. In the gut (right), cAMP/PKA signalling in enteroendocrine L-cells enhances GLP-1 secretion, lowering glucagon, augmenting glucose-dependent insulin secretion, and delaying gastric emptying [55]. The net effect is insulin-independent glycaemic control with β-cell preservation (centre). The depicted pathways derive predominantly from in vitro and rodent studies; human evidence is limited to small, short-term studies. Solid arrows indicate the direction of the signalling step or downstream effect described. ↑ increase, ↓ decrease Image credit: created by the authors using BioRender (2026); https://BioRender.com/oor2pii

Rationale for combining ABA+BAIBA

The case for combining ABA and BAIBA rests on mechanistic complementarity. Each molecule acts on targets the other does not, and the two converge on shared nodes such as AMPK, PGC-1α and mitochondrial function. Figure 5 contrasts the receptor-level pathways engaged by each metabolite and shows where their downstream effects converge. Table 3 maps each molecule against the three therapeutic pillars set out above, together with the level of evidence supporting each mechanism.

**Figure 5:**
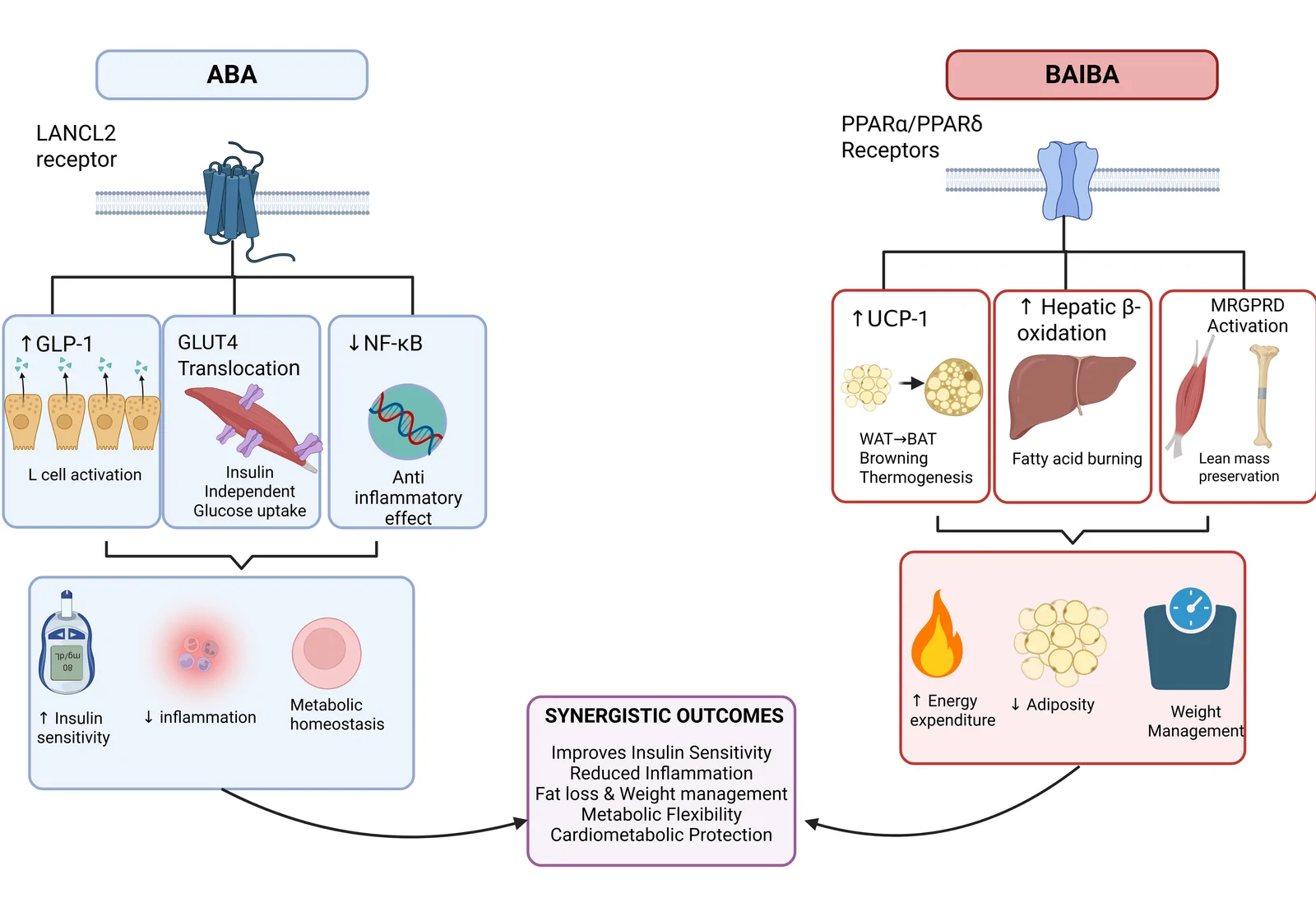
Complementary and synergistic mechanisms of ABA and BAIBA in metabolic regulation BAIBA: β-aminoisobutyric acid; ABA: abscisic acid; LANCL2: lanthionine synthetase C-like protein 2x; BAT: brown adipose tissue; GLP-1: glucagon-like peptide-1; UCP1: uncoupling protein-1; NF-κB: nuclear factor kappa B; MRGPRD: Mas-related G protein-coupled receptor member D; BAT: brown adipose tissue; WAT: white adipose tissue; GLUT-4: insulin-independent glucose transporter type 4; PPARα/PPARδ: peroxisome proliferator-activated receptor alpha and delta Schematic comparing the receptor-level pathways of ABA (left) and BAIBA (right) and their convergent outcomes (centre). ABA signals via LANCL2 to enhance GLP-1 secretion, drive GLUT4 translocation, and suppress NF-κB-driven inflammation [44,55,59], while BAIBA acts via PPARα/PPARδ to upregulate UCP-1 and adipose browning, increase hepatic β-oxidation, and activate MRGPRD for lean mass and bone preservation [25,42,43]. Acting on largely non-overlapping targets, the two are proposed to converge on improved insulin sensitivity, reduced inflammation, fat loss, metabolic flexibility, and cardiometabolic protection; these convergent effects are hypothesised and have not been demonstrated for the combination. Solid arrows indicate the direction of the signalling step or downstream effect described. ↑ increase, ↓ decrease Image credit: created by the authors using BioRender (2026); https://BioRender.com/btrxmwd

Three features distinguish the combination conceptually from incretin monotherapy. First, it targets fat quality and thermogenesis rather than appetite, so any weight lost would be expected to be biased toward dysfunctional WAT. Second, it is intended to support muscle and bone during fat loss rather than accepting their depletion as collateral. Third, its glucose-lowering arm is largely insulin-independent, which could circumvent the β-cell constraint that limits insulin-secretion-dependent strategies in South Asians. Both components are endogenous human metabolites, and an oral combination may offer tolerability and adherence advantages over injectable peptides in chronic metabolic care. 

**Table 3:** Mechanistic complementarity of BAIBA and ABA against the three therapeutic pillars, with supporting level of evidence ABA: abscisic acid; AMPK: adenosine monophosphate-activated protein kinase; BAIBA: β-aminoisobutyric acid; BMD: bone mineral density; GLP-1: glucagon-like peptide-1; GLUT4: glucose transporter type 4; MRGPRD: Mas-related G protein-coupled receptor member D; PGC-1α: peroxisome proliferator-activated receptor gamma coactivator 1-alpha; PPAR: peroxisome proliferator-activated receptor; UCP-1: uncoupling protein-1; UCP1: uncoupling protein-1 gene; WAT: white adipose tissue The three-pillar model is a conceptual framework and has not been validated as a composite clinical endpoint. Evidence levels are defined as follows: preclinical refers to in vitro and/or animal data; human observational refers to association studies of circulating metabolite levels; and early human refers to small, uncontrolled, or short-term interventional studies. No randomised controlled trial of either component on these outcomes, or of the combination, has been published. Source: BAIBA [25,42,43,49-53]; ABA [44,45,55-59]; UCP1 promoter variants [11-15]

Therapeutic pillar	BAIBA	Evidence (BAIBA)	ABA	Evidence (ABA)
WAT-selective (thermogenic) loss	Primary driver: UCP-1 browning via PPARα; may offset UCP1-variant effect	Preclinical (cell, murine); human observational	Supportive: AMPK/PGC-1α raises mitochondrial oxidative capacity	Preclinical
Musculoskeletal preservation	Muscle via AMPK-PPARδ; osteocyte survival via MRGPRD (estrogen-independent)	Preclinical; human observational (BMD)	Muscle via AMPK/PGC-1α/Sirt1; GLUT4 supports muscle energetics	Preclinical (cell)
β-cell-sparing glucose control	Indirect: improved insulin sensitivity with browning	Preclinical	Primary driver: insulin-independent GLUT4 uptake; GLP-1 support; anti-inflammatory via PPARγ	Preclinical; small early human studies

The case for the combination therefore rests on the component data summarised above. No randomised trial of ABA plus BAIBA has been published, and the combination's effects are inferred from separate preclinical and early human studies together with the preliminary open-label experience described below. Potential interactions have not been studied. Because both molecules activate AMPK, their effects on this node could be additive, or they could saturate. Co-administration with other AMPK-engaging or glucose-lowering drugs, such as metformin, sulfonylureas, or insulin, has not been evaluated for pharmacodynamic interaction or hypoglycaemic risk. Both molecules occur naturally in humans. ABA is present in fruits and vegetables, including figs and apricots, and is produced endogenously by human cells [54,56,60]. BAIBA is generated endogenously from thymine and valine catabolism, and its circulating levels are influenced by exercise and by genetic variation in the enzyme alanine-glyoxylate aminotransferase 2 (AGXT2) [42,46].

Early clinical experience

Preliminary clinical experience with an oral ABA plus BAIBA combination comes from DUAL-ACT, an investigator-led, open-label study conducted in India and published as an interim analysis [61]. In 30 adults with obesity treated for 12 weeks, the mean body weight fell from approximately 105.7 kg to 97.2 kg, a reduction of about 8.4 kg or 8%, and no serious adverse events, hypoglycaemic episodes, or treatment discontinuations were reported. Body weight was the principal outcome of this interim report, and glycaemic, insulin, and body-composition outcomes will have to be reported in the full analysis. Because the analysis is interim, small and without a control group, it cannot distinguish a treatment effect from lifestyle co-intervention, placebo response or regression to the mean. It does not establish efficacy or safety and should be regarded as hypothesis-generating only.

Population, safety, and regulatory considerations in India

If its efficacy is confirmed in controlled trials, an ABA plus BAIBA approach could be relevant to several groups that are poorly served by incretin therapy. These include metabolically obese normal-weight adults who fall outside anti-obesity drug labels; peri- and post-menopausal women, in whom bone and muscle preservation are priorities and estrogen-independent osteocyte protection would be attractive; women with PCOS-related sub-fertility, for whom pre-conception weight-loss pharmacotherapy is constrained; and frail or older adults, in whom further lean-mass loss is hazardous and who were under-represented in the pivotal incretin trials [1,6,8,20,35]. The small human studies of ABA did not report adverse effects with microgram doses given for up to 75 days [56,57]. Long-term safety data for either molecule at supplemental doses, and for the combination, are lacking.

Regulatory frameworks for weight-reduction products continue to anchor efficacy to sustained reduction in body weight rather than to change in body composition or metabolic function. This is itself part of the mismatch described in this review, and it explains why weight has remained the conventional primary endpoint [62]. In India, the regulatory pathway for an ABA plus BAIBA combination depends on its composition, source, and intended claims. Products containing these metabolites may be regulated as health supplements or nutraceuticals under the Food Safety and Standards (Health Supplements, Nutraceuticals, Food for Special Medical Purpose, and Prebiotic and Probiotic Food) Regulations, 2022, which do not permit disease-treatment claims. A product intended to treat obesity or T2D would instead require evaluation as a new drug under the New Drugs and Clinical Trials Rules, 2019. This distinction determines the evidentiary standard, trial design, and permissible claims.

Limitations and future directions

Several limitations of this review should be considered when interpreting its conclusions. As a narrative review, it draws on literature selected for mechanistic relevance and applicability to South Asian populations rather than through a systematic search. It did not include a formal risk-of-bias assessment, and some degree of selection bias cannot be excluded.

The evidence base for each component is still developing and is currently weighted toward preclinical work. The effects of BAIBA on adipose browning, muscle and osteocyte survival have been characterised mainly in cell and rodent models. Human data so far consist of observational associations with bone density and cardiometabolic risk. Human studies of ABA, while encouraging, have been small and of short duration, and they used a range of preparations at microgram doses. A randomised controlled trial of the ABA plus BAIBA combination has not yet been reported. Clinical experience to date comes from an interim, open-label, single-arm analysis of 30 participants over 12 weeks.

Further work is also needed to define the pharmacokinetics, optimal dose, ratio, and formulation of the combination, to establish long-term safety, and to explore potential interactions with other AMPK-engaging or glucose-lowering therapies. South Asian populations are diverse in diet, genetics, and body composition, and genetic variation influences circulating BAIBA levels. The UCP1 variant data also come from broader Asian cohorts rather than from TOFI individuals specifically. 

Pharmacokinetic and dose-finding studies would provide the foundation for randomised, double-blind, placebo-controlled trials with body-composition primary endpoints, such as DXA- or MRI-derived visceral adipose tissue and appendicular lean mass. Secondary endpoints could include BMD and bone turnover markers; β-cell function assessed by HOMA-B, C-peptide and OGTT-derived disposition index; HbA1c, fasting glucose and fasting insulin; and muscle strength and physical performance. Prespecified subgroups of particular interest include metabolically obese normal-weight adults, post-menopausal women, and older adults at risk of sarcopenia. Add-on designs alongside GLP-1 RAs could explore whether the combination helps preserve lean and bone mass during incretin therapy, and head-to-head designs could compare body-composition outcomes directly. If it is supported by controlled evidence, a body-composition-centred approach could also encourage wider adoption of body-composition and functional endpoints, alongside body weight, in obesity and T2D drug development.

## Conclusions

In a large proportion of South Asians, the TOFI phenotype combines low muscle mass, low bone density, and early decline in β-cell function. For these patients, the most relevant therapeutic question may be not how much weight can be removed, but how the underlying metabolic architecture can be restored. Incretin-based therapies, for all their efficacy, may be an imperfect match. They can reduce lean and bone mass that such patients have little reserve to spare; their glucose-lowering action depends on declining β-cell function; and they are licensed for a body size many of these patients never reach. A combination of ABA and BAIBA may offer a mechanistically plausible, body-composition-preserving approach. In this approach, BAIBA would promote browning of fat and support muscle and bone, while ABA would promote glucose disposal largely independent of β-cell secretory capacity. The supporting evidence is currently preclinical and early human, and preliminary open-label data are limited. No randomised controlled trial of the combination has yet been reported. It warrants rigorous randomised evaluation in South Asian populations, with body-composition, glycaemic, and β-cell function endpoints and, post-validation, it could encourage the wider use of body-composition and functional outcomes, alongside body weight, in obesity and T2D drug development.

## References

[1] Rao AR, Bhagwasia M, Singh V (2025). Prevalence and determinants of sarcopenia among older adults in India: insights from the longitudinal aging study in India. J Am Geriatr Soc.

[2] Cho EJ, Choi Y, Jung SJ, Kwak HB (2022). Role of exercise in estrogen deficiency-induced sarcopenia. J Exerc Rehabil.

[3] Yajnik CS, Yudkin JS (2004). The Y-Y paradox. Lancet.

[4] Kodama K, Tojjar D, Yamada S, Toda K, Patel CJ, Butte AJ (2013). Ethnic differences in the relationship between insulin sensitivity and insulin response: a systematic review and meta-analysis. Diabetes Care.

[5] Li L, Zou X, Huang Q, Han X, Zhou X, Ji L (2021). Do East Asians with normal glucose tolerance have worse β-cell function? A meta-analysis of epidemiological studies. Front Endocrinol (Lausanne).

[6] Anjana RM, Unnikrishnan R, Deepa M (2023). Metabolic non-communicable disease health report of India: the ICMR-INDIAB national cross-sectional study (ICMR-INDIAB-17). Lancet Diabetes Endocrinol.

[7] Anjana RM, Pradeepa R, Das AK (2014). Physical activity and inactivity patterns in India-results from the ICMR-INDIAB study (Phase-1) [ICMR-INDIAB-5]. Int J Behav Nutr Phys Act.

[8] Wilding JP, Batterham RL, Calanna S (2021). Once-weekly semaglutide in adults with overweight or obesity. N Engl J Med.

[9] Look M, Dunn JP, Kushner RF (2025). Body composition changes during weight reduction with tirzepatide in the SURMOUNT-1 study of adults with obesity or overweight. Diabetes Obes Metab.

[10] Golozoubova V, Cannon B, Nedergaard J (2006). UCP1 is essential for adaptive adrenergic nonshivering thermogenesis. Am J Physiol Endocrinol Metab.

[11] Chathoth S, Ismail MH, Vatte C (2018). Association of uncoupling protein 1 (UCP1) gene polymorphism with obesity: a case-control study. BMC Med Genet.

[12] Nagai N, Sakane N, Tsuzaki K, Moritani T (2011). UCP1 genetic polymorphism (-3826 A/G) diminishes resting energy expenditure and thermoregulatory sympathetic nervous system activity in young females. Int J Obes (Lond).

[13] Jia JJ, Tian YB, Cao ZH (2010). The polymorphisms of UCP1 genes associated with fat metabolism, obesity and diabetes. Mol Biol Rep.

[14] Lee KH, Chai VY, Kanachamy SS, Say YH (2015). Association of UCP1 -3826A/G and UCP3 -55C/T gene polymorphisms with obesity and its related traits among multi-ethnic Malaysians. Ethn Dis.

[15] Brondani LA, de Souza BM, Assmann TS, Bouças AP, Bauer AC, Canani LH, Crispim D (2014). Association of the UCP polymorphisms with susceptibility to obesity: case-control study and meta-analysis. Mol Biol Rep.

[16] Lear SA, Kohli S, Bondy GP, Tchernof A, Sniderman AD (2009). Ethnic variation in fat and lean body mass and the association with insulin resistance. J Clin Endocrinol Metab.

[17] Ntuk UE, Celis-Morales CA, Mackay DF, Sattar N, Pell JP, Gill JM (2017). Association between grip strength and diabetes prevalence in black, South-Asian, and white European ethnic groups: a cross-sectional analysis of 418 656 participants in the UK Biobank study. Diabet Med.

[18] Rajan TV, Kerstetter J, Feinn R, Kenny A (2014). Evidence for low androgenicity among Indian (South Asian) men. Aging Male.

[19] Lai KM, Gonzalez M, Poueymirou WT (2004). Conditional activation of akt in adult skeletal muscle induces rapid hypertrophy. Mol Cell Biol.

[20] Babhulkar S, Seth S (2021). Prevalence of osteoporosis in India: an observation of 31,238 adults. Int J Res Orthop.

[21] Ji MX, Yu Q (2015). Primary osteoporosis in postmenopausal women. Chronic Dis Transl Med.

[22] Marwaha RK, Tandon N, Garg MK (2011). Bone health in healthy Indian population aged 50 years and above. Osteoporos Int.

[23] Aparna P, Muthathal S, Nongkynrih B, Gupta SK (2018). Vitamin D deficiency in India. J Family Med Prim Care.

[24] Lu L, Tian L (2023). Postmenopausal osteoporosis coexisting with sarcopenia: the role and mechanisms of estrogen. J Endocrinol.

[25] Kitase Y, Vallejo JA, Gutheil W (2018). β-aminoisobutyric acid, L-BAIBA, is a muscle-derived osteocyte survival factor. Cell Rep.

[26] Wysham C, Shubrook J (2020). Beta-cell failure in type 2 diabetes: mechanisms, markers, and clinical implications. Postgrad Med.

[27] Jones AG, McDonald TJ, Shields BM, Hill AV, Hyde CJ, Knight BA, Hattersley AT (2016). Markers of β-cell failure predict poor glycemic response to GLP-1 receptor agonist therapy in type 2 diabetes. Diabetes Care.

[28] Cornell S (2020). A review of GLP-1 receptor agonists in type 2 diabetes: a focus on the mechanism of action of once-weekly agents. J Clin Pharm Ther.

[29] Ko D, Kim DH, Tan FX (2026). Suboptimal therapeutic responses associated with semaglutide in type 2 diabetes and obesity: a meta-analysis of randomized controlled trials. Cell Rep Med.

[30] Hansen MS, Wölfel EM, Jeromdesella S (2024). Once-weekly semaglutide versus placebo in adults with increased fracture risk: a randomised, double-blinded, two-centre, phase 2 trial. EClinicalMedicine.

[31] Liu Y, Walzer D, Schmitz S, Shukla A, Kashyap SR, D’Angelo D (2025). MON-817 association of semaglutide and tirzepatide use on bone density and fracture risk in obese patients with and without diabetes. J Endocr Soc.

[32] Cheng L, Hu Y, Li YY (2019). Glucagon-like peptide-1 receptor agonists and risk of bone fracture in patients with type 2 diabetes: a meta-analysis of randomized controlled trials. Diabetes Metab Res Rev.

[33] Price SA, Nankervis A (2025). Considering the use of GLP-1 receptor agonists in women with obesity prior to pregnancy: a narrative review. Arch Gynecol Obstet.

[34] Alshehri FS (2025). New developments in GLP-1 agonist therapy for gestational diabetes: Systematic review on liraglutide, semaglutide, and exenatide from ClinicalTrials.gov. Medicine (Baltimore).

[35] Minis E, Stanford FC, Mahalingaiah S (2023). Glucagon-like peptide-1 receptor agonists and safety in the preconception period. Curr Opin Endocrinol Diabetes Obes.

[36] West S, Scragg J, Aveyard P (2026). Weight regain after cessation of medication for weight management: systematic review and meta-analysis. BMJ.

[37] Wilding JP, Batterham RL, Davies M (2022). Weight regain and cardiometabolic effects after withdrawal of semaglutide: the STEP 1 trial extension. Diabetes Obes Metab.

[38] Beavers KM, Lyles MF, Davis CC, Wang X, Beavers DP, Nicklas BJ (2011). Is lost lean mass from intentional weight loss recovered during weight regain in postmenopausal women?. Am J Clin Nutr.

[39] Qaisar R, Khan IU, Rehman AU, Iqbal MS, Ahmad F, Karim A (2026). Semaglutide use is associated with neuromuscular junction degradation in older adults with type II diabetes mellitus. Br J Clin Pharmacol.

[40] Heymsfield SB, Aronne LJ, Montgomery P (2026). Bimagrumab plus semaglutide alone or in combination for the treatment of obesity: a randomized phase 2 trial. Nat Med.

[41] (2026). Dose-finding study evaluating effect on body composition of enobosarm in patients taking a GLP-1 for chronic weight mgmt (QUALITY). https://clinicaltrials.gov/study/NCT06282458.

[42] Roberts LD, Boström P, O'Sullivan JF (2014). β-Aminoisobutyric acid induces browning of white fat and hepatic β-oxidation and is inversely correlated with cardiometabolic risk factors. Cell Metab.

[43] Jung TW, Hwang HJ, Hong HC, Yoo HJ, Baik SH, Choi KM (2015). BAIBA attenuates insulin resistance and inflammation induced by palmitate or a high fat diet via an AMPK-PPARδ-dependent pathway in mice. Diabetologia.

[44] Magnone M, Emionite L, Guida L (2020). Insulin-independent stimulation of skeletal muscle glucose uptake by low-dose abscisic acid via AMPK activation. Sci Rep.

[45] Spinelli S, Begani G, Guida L (2021). LANCL1 binds abscisic acid and stimulates glucose transport and mitochondrial respiration in muscle cells via the AMPK/PGC-1α/Sirt1 pathway. Mol Metab.

[46] Tanianskii DA, Jarzebska N, Birkenfeld AL, O'Sullivan JF, Rodionov RN (2019). Beta-aminoisobutyric acid as a novel regulator of carbohydrate and lipid metabolism. Nutrients.

[47] Jagtap U, Paul A (2023). UCP1 activation: Hottest target in the thermogenesis pathway to treat obesity using molecules of synthetic and natural origin. Drug Discov Today.

[48] Kaisanlahti A, Glumoff T (2019). Browning of white fat: agents and implications for beige adipose tissue to type 2 diabetes. J Physiol Biochem.

[49] Narkar VA, Downes M, Yu RT (2008). AMPK and PPARdelta agonists are exercise mimetics. Cell.

[50] Vallejo JA, Kitase Y, Ganesh T (2025). L-β-aminoisobutyric acid (L-BAIBA) in combination with voluntary wheel running exercise enhances musculoskeletal properties in middle-age male mice. Aging (Albany NY).

[51] Hamrick MW, McGee-Lawrence ME (2018). Blocking bone loss with l-BAIBA. Trends Endocrinol Metab.

[52] Huang ZW, Yu YP, He XR (2025). Exercise suppresses osteoclastogenesis by increasing the secretion of muscle-derived L-β-aminoisobutyric acid. J Sport Health Sci.

[53] Zhu XW, Ding K, Dai XY, Ling WQ (2018). β-aminoisobutyric acid accelerates the proliferation and differentiation of MC3T3-E1 cells via moderate activation of ROS signaling. J Chin Med Assoc.

[54] Bruzzone S, Moreschi I, Usai C (2007). Abscisic acid is an endogenous cytokine in human granulocytes with cyclic ADP-ribose as second messenger. Proc Natl Acad Sci U S A.

[55] Bruzzone S, Magnone M, Mannino E (2015). Abscisic acid stimulates glucagon-like peptide-1 secretion from L-cells and its oral administration increases plasma glucagon-like peptide-1 levels in rats. PLoS One.

[56] Magnone M, Ameri P, Salis A (2015). Microgram amounts of abscisic acid in fruit extracts improve glucose tolerance and reduce insulinemia in rats and in humans. FASEB J.

[57] Magnone M, Leoncini G, Vigliarolo T, Emionite L, Sturla L, Zocchi E, Murialdo G (2018). Chronic intake of micrograms of abscisic acid improves glycemia and lipidemia in a human study and in high-glucose fed mice. Nutrients.

[58] Leber A, Hontecillas R, Tubau-Juni N, Zoccoli-Rodriguez V, Goodpaster B, Bassaganya-Riera J (2020). Abscisic acid enriched fig extract promotes insulin sensitivity by decreasing systemic inflammation and activating LANCL2 in skeletal muscle. Sci Rep.

[59] Bassaganya-Riera J, Guri AJ, Lu P (2011). Abscisic acid regulates inflammation via ligand-binding domain-independent activation of peroxisome proliferator-activated receptor gamma. J Biol Chem.

[60] Zocchi E, Hontecillas R, Leber A (2017). Abscisic acid: a novel nutraceutical for glycemic control. Front Nutr.

[61] Rukadikar K, Samant R (2026). A dual-mechanism oral therapy targeting UCP-1 and GLP-1 pathways for obesity management: interim results from the DUAL-ACT trial. Int J Endocrinol Res.

[62] (2026). Obesity and overweight: developing drugs and biological products for weight reduction. https://www.fda.gov/regulatory-information/search-fda-guidance-documents/obesity-and-overweight-developing-drugs-and-biological-products-weight-reduction..

